# The effect of *in vitro* consecutive passages and culture medium on the genetic variations in BCG Pasteur 1173P2 vaccine

**DOI:** 10.1371/journal.pone.0280294

**Published:** 2023-01-23

**Authors:** Mahla Asadian, Seyed Mehdi Hassanzadeh, Azadeh Safarchi, Masoumeh Douraghi

**Affiliations:** 1 Division of Microbiology, Department of Pathobiology, School of Public Health, Tehran University of Medical Sciences, Tehran, Iran; 2 BCG Vaccine Production Plant, Pasteur Institute of Iran, Karaj, Iran; 3 School of Biotechnology and Biomolecular Sciences, University of New South Wales, Sydney, Australia; Translational Health Science and Technology Institute, INDIA

## Abstract

Since the introduction of the Bacillus Calmette–Guérin (BCG) vaccine, the genomes of vaccine strains have undergone variations due to repeated passages in different laboratories and vaccine production facilities. Genetic variations have been considered as one of the effective factors in the BCG variable protective efficacy. Consecutive subcultures have been shown to play an essential role in causing genetic variations in several microorganisms, including *Mycobacterium bovis* BCG. Therefore, the world health organization (WHO) recommendation to limit the passages of master seed lot in the BCG vaccine production should be considered. Besides, the role of other external variables such as quality of the raw ingredients of the culture media, the type of the culture medium and the cultivation methods in the vaccine production has been poorly studied. Here, the effect of passages and culture medium on genetic variations in a BCG seed lot was investigated during a year. The findings of this study revealed a total of 19 variants compared to seed lot while the passages were more than the number recommended by WHO. The first culture of seed lot in the Sauton broth and Middlebrook 7H9 media, and the last subculture in Sauton broth had the least and the most variants, respectively. The observation of the higher number of variants in the last cultures on Sauton broth and Middlebrook 7H9 in comparison to the first and the middle cultures may indicate the effect of passages on the genetic variations in BCG. Additionally, more variants in BCG grown in the Sauton broth do not necessarily represent the greater ability of this medium to cause genetic mutations. For a better conclusion, it is required to examine the medium components as independent variables.

## Introduction

Bacillus Calmette-Guérin (BCG) refers to a family of attenuated strains derived from an isolate of *Mycobacterium bovis* by serial subcultures on potato slices soaked in ox bile and glycerol [1, 2]. Although BCG is more widely administered than other vaccines worldwide, variability in its protective efficacy in preventing pulmonary tuberculosis has been reported by different studies [3, 4]. Several reasons including differences in the vaccine preparation conditions can be associated with these variable outcomes [5–10]. Following the continuous subcultures of the initial BCG vaccine at different laboratories worldwide, various vaccine strains appeared with phenotypic and genotypic differences. Owing to the maintenance of these strains by successive cultures until the introduction of freeze-dried seed lots in the 1960s, significant variations occurred in each strain’s genome [7, 11]. To prevent the accumulation of variations, the world health organization (WHO) proposed that the vaccine should not be obtained from a culture with more than 12 passages of the lyophilized master seed lot [12].

Many studies have attributed the attenuation of the original virulent strain to various genetic mutations in its genome that occurred due to sequential subcultures [1, 13]. Considering the genetic variations that have been shared in all vaccine strains compared to their parent and the exclusive genetic variations of each strain [1], it can be concluded that the *in vitro* consecutive passages may be a substantial factor in the occurrence of genetic variations. The effect of *in vitro* continuous passages on mutations affecting the virulence and/or genetic characteristics of *Mycobacterium tuberculosis* (*M*. *tuberculosis*) and the protective efficacy of BCG vaccine in clinical trials have been reported by several studies [14–20].

While some countries provide their required BCG vaccine from international manufacturers, the other ones produce their vaccine. Danish 1331, Russia BCG-1, Tokyo 172–1, and Moreau-RJ strains have been represented by the WHO Expert Committee on Biological Standardization (ECBS) as BCG reference strains [21, 22]. However, there is no standardized method or particular culture medium for the production of BCG vaccines. The probability that different culture media may also affect the phenotypic and genotypic characteristics, and subsequently, the clinical efficacy of BCG vaccines needs further investigation [8, 23]. Among the lyophilized vaccine master seed lots, only the master seed lot pertains to Pasteur strain originates from a single colony and can be called a strain literally [24]. This seed lot is used to produce the BCG vaccine in Iran [25]. This study aimed to seek the effect of external variables on genetic variations in a BCG vaccine strain. To achieve this goal, the occurrence of genetic variations in the BCG Pasteur 1173P2 was investigated by *in vitro* serial passages of a lyophilized seed lot within a year on different culture media.

## Materials and methods

### Culture of seed lot and sample selection

The seed lot for BCG Pasteur 1173P2 (seed lot C) was obtained from the Pasteur Institute (France, Paris) and passaged by the method for vaccine production within a year (May 2019 to May 2020) continuously. Briefly, the freeze-dried seed lot was inoculated in the Sauton Potato and Sauton broth which are the most commonly used media in the vaccine production according to a recipe as previously described [26], and Middlebrook 7H9 (Sigma-Aldrich) supplemented with 10% Albumin-Dextrose-Catalase (Sigma-Aldrich) and 0.4% glycerol which is reported to be the most commonly used medium in the laboratory studies. Considering that the growth rate of BCG was different on each media, the time intervals of passages on Sauton Potato, Sauton broth, and Middlebrook 7H9 was every 18, 10, and 21 days, respectively. Bacterial suspensions from the first, middle (after six months), and last culture on all three media were collected. The samples included in this study were named as shown in Table 1.

**Table 1:** Selected samples of the serial passages.

Sample name	Description
C^a^SP^b^F^c^	First culture of seed lot C on Sauton potato culture medium
CSB^d^F	First culture of seed lot C on Sauton broth culture medium
CMB^e^F	First culture of seed lot C on Middlebrook 7H9 culture medium
CSPM^f^	Middle culture of seed lot C on Sauton potato culture medium
CSBM	Middle culture of seed lot C on Sauton broth culture medium
CMBM	Middle culture of seed lot C on Middlebrook 7H9 culture medium
CSPL^g^	Last culture of seed lot C on Sauton potato culture medium
CSBL	Last culture of seed lot C on Sauton broth culture medium
CMBL	Last culture of seed lot C on Middlebrook 7H9 culture medium

^a^ Seed lot C

^b^ Sauton potato

^c^ First

^d^ Sauton broth

^e^ Middlebrook 7H9

^f^ Middle

^g^ Last

### Preparation of genomic DNA and whole genome sequencing

Molecular identity of the samples was determined for dried BCG vaccines using the RD14 and DU1 regions [27, 28]. The following PCR primers were used: RD14; F: 5′-CTGGTACACCTGGGGAATCTGG-3′, R: 5′-CAGGGTTGAAGGAATGCGTGTC-3′ and DU1; F: 5′-ACGGTCGGTGTCGTCTAAGT-3′, R: 5′-AGAACTGCAGGGGTGGTACA-3′ [27, 28]. PCR conditions were as follows: initial denaturation at 94°C for 10 min, followed by 30 cycles of 1 min at 94°C, 1 min at 50°C, 2 min at 72°C and then a final extension at 72°C for 10 min [27]. Genomic DNA was extracted from the selected cultures using the CTAB method [29]. The library preparation was conducted using modified Nextera Flex protocol (Hackflex) [30] and genomes were sequenced using the Illumina NovaSeq 6000 platform with an average coverage of 94-fold at University of Technology Sydney (Sydney, Australia). The raw reads were submitted to GenBank with BioProject and BioSample numbers as indicated in Table 2.

**Table 2:** Properties of the selected samples.

Sample name	BioSample number	SRA number	BioProject number
CSPF	SAMN21156925	SRR15676439	PRJNA759141
CSBF	SAMN17277795	SRR13399343	PRJNA691088
CMBF	SAMN17277796	SRR13399342	
CSPM	SAMN21156926	SRR15676438	PRJNA759141
CSBM	SAMN21156927	SRR15676437	
CMBM	SAMN21156928	SRR15676436	
CSPL	SAMN21156929	SRR15676435	
CSBL	SAMN21156930	SRR15676434	
CMBL	SAMN21156931	SRR15676433	

### Genome assembly and analysis

Raw paired-end reads were processed to trim adapter sequences and low-quality ends using Trimmomatic (v0.39) [31] and checked for quality using FastQC (v0.11.9) [32]. The short reads were *de novo* assembled with Shovill pipeline [https://github.com/tseemann/shovill] in combination with SPAdes (v3.13.1) [33] and contigs shorter than 1 Kbp were excluded prior to being evaluated by QUAST (v5.0.2) [34, 35]. The generated contigs were aligned and rearranged to create comparable data using Mauve aligner (v20150226) as described previously [36], and then, annotated using the RAST server [37]. Furthermore, the raw short reads were mapped to the reference genome of BCG Pasteur 1173P2 (accession number: NC_008769.1) using Burrows-Wheeler Aligner (BWA) (v0.7.17) [38]. The mapping quality was assessed using Qualimap (v2.2.1) [39]. Calling of variants was performed by SAMtools (v0.1.19) with mpileup command [40] and Mauve (v20150226) software [36]; Common variants detected by both methods were retained.

## Results

### Serial passages of the seed lot

Following the consecutive passages of the seed lot, 19, 34 and 16 passages were performed on the Sauton potato, Sauton broth and Middlebrook 7H9 media, respectively. During the annual passages, no change was observed in growth rate and phenotypic characteristics of the grown bacteria on all three media. All selected samples were confirmed as BCG Pasteur 1173P2 using clump appearance on Sauton potato, acid-fast staining, deletion of the RD14 (S1 Fig), and the presence of DU1 region (S2 Fig).

### Genetic stability of the BCG Pasteur 1173P2 during the consecutive passages

Scaffolds annotation for the sequences showed the average of total genes 4298 including 4250 coding sequences (CDS), three rRNA genes (5S, 16S and 23S), 44 tRNA genes, two ncRNA genes (*rnp*B and *ffs*) and a tmRNA gene (*ssr*A). Genetic variations including single nucleotide polymorphisms (SNPs) and insertions and deletions (InDels) less than 100 bp were identified by mapping as well as alignment with the reference strain genome. Obtained variants from the variant call output format (VCF) files with the read depth and quality less than 75% and 30 were removed, respectively [41]. In total, a catalog of 19 true variants including 14 SNPs and five InDels was identified (Tables 3 and 4).

**Table 3:** List of SNPs in the selected samples from the seed lot.

Position in BCG Pasteur 1173P2	SNP	Locus_tag	CDS	Product	Amino acid substitution	First			Middle			Last		
						culture			culture			culture		
						(1-1-1)^a^			(9-17-8)^b^			(19-34-16)^c^		
						CSPF	CSBF	CMBF	CSPM	CSBM	CMBM	CSPL	CSBL	CMBL
416686	G -> A	RS01830	416100..416870	Glycerophosphodiester phosphodiesterase	Alanine -> Valine	-	-	-	-	-	-	+	+	-
705623	G -> A	RS03180	702887..706798	PE family protein	Histidine -> Histidine	+	-	-	+	+	+	+	+	+
705626	G -> C				Alanine -> Alanine	+	-	-	+	+	+	+	+	+
1344671	C -> G	Intergenic	-	-	-	+	+	+	+	+	+	+	+	+
1344672	G -> C	Intergenic	-	-	-	+	+	+	+	+	+	+	+	+
2287780	C -> G	RS10645	2278183..2290638	Type I polyketide synthase	Proline -> Glycine	-	-	-	+	-	-	+	+	+
2287781	G -> C				Proline -> Glycine	-	-	-	+	-	-	+	+	+
2287782	G -> C				Proline -> Glycine	-	-	-	+	-	-	+	+	+
2287789	G -> T				Asparagine -> Arginine	-	-	-	+	-	-	+	+	+
2287790	T -> C				Asparagine -> Arginine	-	-	-	+	-	-	+	+	+
2287800	A -> G				Leucine -> Leucine	-	-	-	+	-	-	+	+	+
2764157	T -> A	RS12955	2761247..2764687	LuxR family transcriptional regulator	Threonine -> Threonine	+	+	+	+	+	+	+	+	+
3907860	A -> G	RS18320	3906066..3910184	PE family protein	Asparagine -> Aspartate	-	-	-	+	+	+	-	+	-
3908180	C -> T				Glycine -> Glycine	-	-	-	+	+	+	-	+	-

^a^ Number of the first culture on Sauton potato, Sauton broth and Middlebrook 7H9

^b^ Number of the middle culture on Sauton potato, Sauton broth and Middlebrook 7H9

^c^ Number of the last culture on Sauton potato, Sauton broth and Middlebrook 7H9

**Table 4:** List of InDels in the selected samples from the seed lot.

Position in BCG Pasteur 1173P2	Sequence (REF^a^)	Sequence (ALT^b^)	Insertion/ Deletion	Number of nucleotides	Locus_tag	CDS	Product	First			Middle			Last		
								culture			culture			culture		
								CSPF	CSBF	CMBF	CSPM	CSBM	CMBM	CSPL	CSBL	CMBL
2002384	C	CCGG	Insertion	3 (CGG)	RS09290 (Pseudogene)	2000361..2003050	PE family protein	+	-	-	+	+	+	+	+	+
2766102	C	CCGG	Insertion	3 (CGG)	RS12965 (Pseudogene)	2765061..2770032	PE family protein	+	-	-	+	+	+	+	+	+
3193555	GTCATCACCATCGAACCAGTGATGATCGCAAGCGCGGCGAAGCCGGGCGCAGTCCCCCGCAAGCGGGAGGTGCCCCCAGGT	GT	Deletion	79 (TCATCACCATCGAACCAGTGATGATCGCAAGCGCGGCGAAGCCGGGCGCAGTCCCCCGCAAGCGGGAGGTGCCCCCAGG)	Intergenic	-	-	-	+	+	-	-	+	+	+	+
3833488	GG	GGCCG	Insertion	3 (GCC)	RS17970	3833368..3834786	Bifunctional ADP-dependent NAD(P)H-hydrate dehydratase/NAD(P)H-hydrate epimerase	+	+	+	+	+	+	+	+	+
3854969	GG	GGTCG	Insertion	3 (GTC)	RS18060	3854189..3854974	Cutinase family protein	+	+	+	+	+	+	+	+	+

^a^ Reference

^b^ Alternative

Twelve SNPs in genes (seven non-synonymous and five synonymous) and two SNPs in the intergenic region were identified as two consecutive nucleotide substitutions. Three SNPs were shared among all samples. CSBF and CMBF had the lowest number of SNPs and CSBL had the highest. A non-synonymous SNP in RS01830 encoding a glycerophosphoryl diester phosphodiesterase enzyme was only detected in CSPL and CSBL and may be related to the number of passages. Six SNPs in RS10645 were detected in CSPM, CSPL, CSBL and CMBL. This gene is involved in the polyketide and possibly phytoserol synthesis, and also shows variants in BCG strains compared to the *M*. *tuberculosis* H37Rv.

Of the five InDels identified, four were detected as non-frameshift insertions and one as a 79 bp deletion in the intergenic region. Two InDels in RS17970 and RS18060 were common in all samples. Two insertions in RS09290 and RS12965 that occur within pseudogenes were found in all samples except CSBF and CMBF.

## Discussion

So far, no study has been conducted to investigate the effect of passages and culture medium used in the vaccine production on the occurrence of minor genetic variations in the genome of a BCG vaccine strain. To investigate the WHO requirement for the minimum number of passages (12 passages) from a master seed lot in the BCG vaccine production [12], Pasteur 1173P2 seed lot was cultured for 12 months. Furthermore, the effect of culture medium on the genetic variationswas simultaneously investigated. The Sauton potato and Sauton broth media are the most commonly used media in the BCG vaccine factory and the Middlebrook 7H9 is known as the medium used in research on the BCG vaccine. The current study showed a total of 19 variants. It seems that all identified variants were high quality (true variants). However, due to the presence of repeat regions in PE genes and low accuracy of short read sequencing methods in identifying variants in these regions, it is not possible to verify the variants definitely, as they might be sequencing errors. On the other hand, mycobacteria genome is rich in repeat regions and homopolymers, sequencing errors are more likely to occur in these regions. To achieve more accurate results, it is better to use the short read-based sequencing methods in combination with the long read and increased coverage to minimize the sequencing errors.

The increased genomic variants in bacteria from the last culture in the Sauton broth and Middlebrook 7H9 media compared to the first and the middle cultures may indicate the role of the increased number of passages. While InDels were also higher in the CSPL in comparison to the CSPF and CSPM, SNPs were detected more in the CSPM than CSPL. This findings might be related to the sequencing errors in the PE genes. Moreover, the CSBL showed the highest number of variants compared to the reference strain and the number of passages on Sauton broth was more than the other two media (34 passages); therefore, the increased variants may be ralated to the increased passages. In contrary to the present study, Seki *et al*. investigated the effect of passages during BCG Tokyo 172 production on genetic stability using several specific genomic regions [42]. This study reported no differences between seed lot, 8th and 20th passages of the vaccine product, indicating genetic stability in the tested regions. This study also revealed that the Multiplex PCR is not sufficient to detect the low number of variants, SNPs and short InDels in the vaccine [42], and in this study we decided to use the whole genome sequencing method. In addition, Trovero *et al*. examined the genetic stability of BCG Pasteur 1173P2 after serial passages by examining only some specific genomic regions [43]. This study showed no differences between the 3rd, 6th, 10th, 15th and 20th passages in comparison with the initial culture of seed lot in terms of the studied regions length [43]. In a previous study, the effect of additional cultures on the growth curve of the BCG Moreau and a recombinant BCG strain (rBCG-S1PT) has been evaluated while undergoing the freezing, storage and thawing cycles [44]. Findings of this study on the vaccine strain growth are inconsistent with the present study in which the time intervals between passages were variable on three media and did not follow a specific pattern in decreasing or increasing growth rate.

Since the type of culture medium used in the BCG vaccine production has been considered as an important factor in the production of more effective vaccines against tuberculosis [8], we examined the effect of this factor on the occurrence of genetic variations in the BCG Pasteur 1173P2. Inconsistent to the Venkataswamy’s study, in which obvious differences in the behavior of BCG grown on Sauton broth and Middlebrook 7H9 media were identified [8], we did not find a significant relationship between the identified variants and the type of culture medium used in the serial passages. The higher number of variants in bacteria from the CSBL may be associated to both the increased passages and the type of culture medium, and requires further investigation into more detailed variables. Florio’s study showed that the percentage of live bacilli in Middlebrook 7H9 was significantly lower than of the Sauton broth and Dubos media when exposed to reactive nitrogen mediators [45]. This study also reported that grown BCG in different media had different protein expression patterns and resistance to reactive nitrogen mediators, which is probably due to the antioxidant effect of the medium [45].

Identification of genetic variations in a BCG vaccine strain following the consecutive cultures from a lyophilized seed lot revealed the importance of adhering to the minimum possible passage from a seed lot in the vaccine production (WHO recommendation) to prevent genetic modifications. Genetic variations may have functional effects and may also play a role in the variable protective efficacy of the vaccine. Furthermore, the difference in the number of variants in bacteria grown in different culture media showed that the type of culture medium used in the vaccine production may also be effective in the occurrence of genetic mutations. To examine the impact of this variable more closely, it is required to evaluate the compounds of the culture media separately.

## Supporting information

S1 Fig Confirmation of the vaccine strain as *Mycobacterium tuberculosis* variant bovis BCG str. Pasteur 1173P2 using RD14 primers.
(TIF)

S2 Fig Confirmation of the vaccine strain as *Mycobacterium tuberculosis* variant bovis BCG str. Pasteur 1173P2 using DU1 primers.
(TIF)
