# Supplementary figures and images for: The effect of in vitro consecutive passages and culture medium on the genetic variations in BCG Pasteur 1173P2 vaccine
Source: PLoS One. 2023 Jan 23;18(1):e0280294. doi: 10.1371/journal.pone.0280294 (PMC9870133; doi:10.1371/journal.pone.0280294)

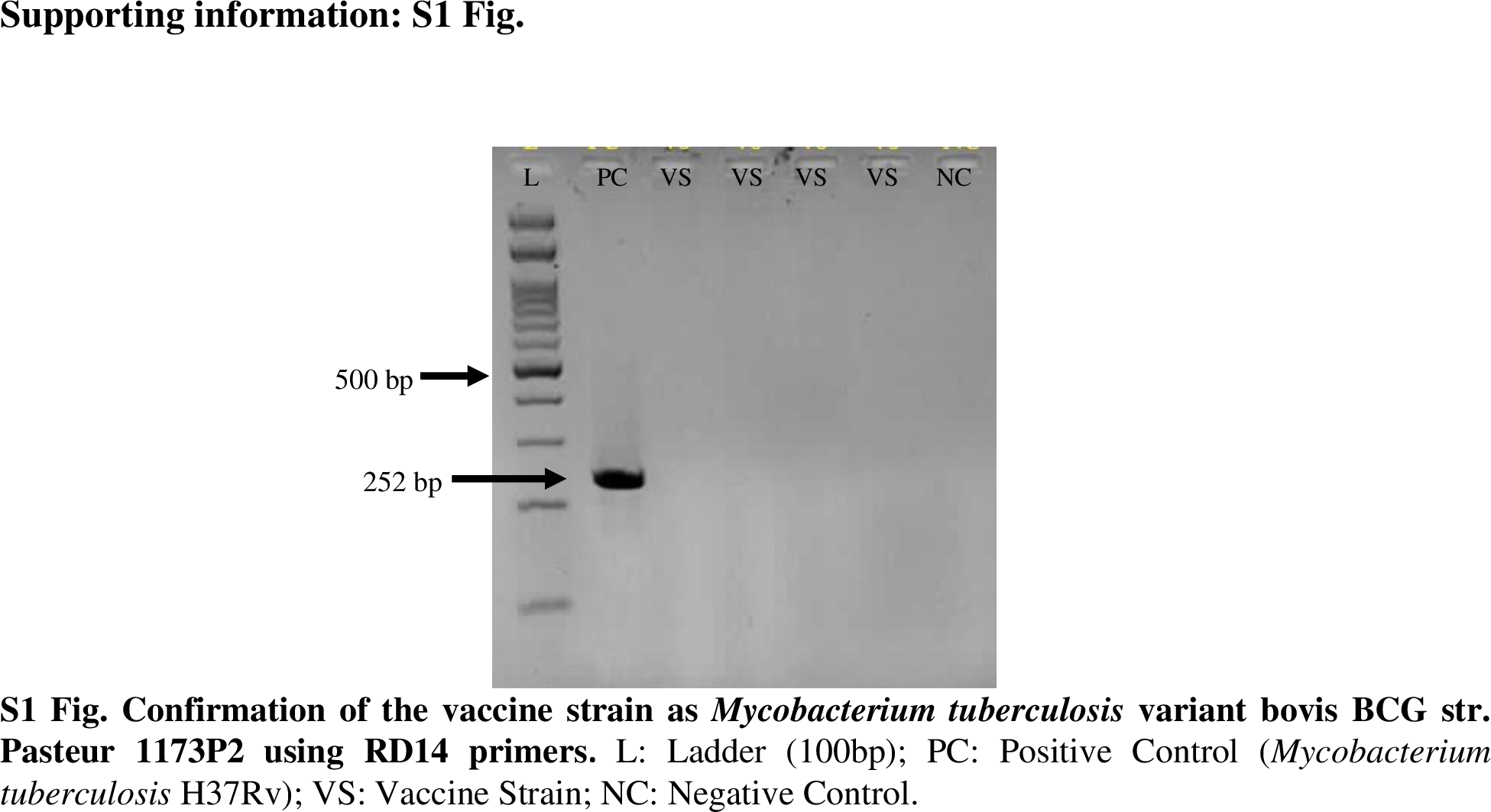

Supplement: S1 Fig — Pasteur 1173P2 using RD14 primers. (TIF) [file pone.0280294.s001.tif]

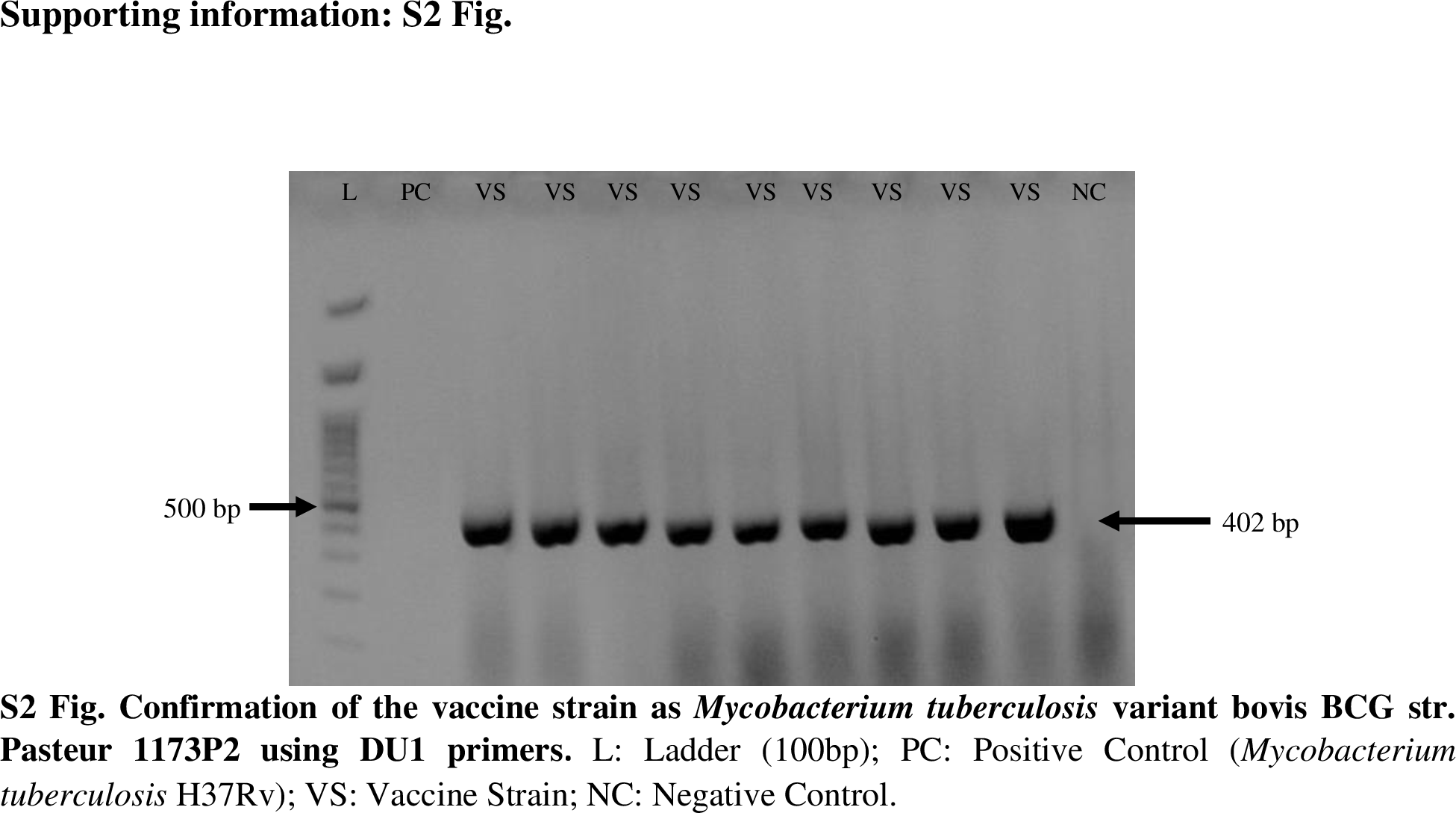

Supplement: S2 Fig — Pasteur 1173P2 using DU1 primers. (TIF) [file pone.0280294.s002.tif]
